# Supplementary material for: Global epistasis in ecosystems arises from resource constraints
Source: bioRxiv. 2026 May 15:2026.05.12.724736. Preprint. [Version 1] doi: 10.64898/2026.05.12.724736 (PMC13192798; doi:10.64898/2026.05.12.724736)
Supplement: Supplement 1 [file media-1.pdf]

SUPPLEMENTARY APPENDIX

GLOBAL EPISTASIS FROM ONE SPECIES MANY RESOURCES

Let $x(t)$ denote biomass; $C_i(t)$ the concentrations of carbon sources $i \in S$; and $N(t)$ the nitrogen concentration. Each carbon source is substitutable with uptake coefficient r_{C_i}, half–saturation K_{C_i}, and yield Y_{C_i}. Nitrogen is essential with r_N, K_N, and yield Y_N.

Under Liebig's law of the minimum, the instantaneous growth rate is

$$\dot{x} = x \, \min\left[\sum_{i \in S} Y_{C_i} \frac{r_{C_i} C_i}{K_{C_i} + C_i} \, , \, Y_N \frac{r_N N}{K_N + N} \right]. \tag{8}$$

Carbon and nitrogen dynamics follow from stoichiometry:

$$\dot{C}_i = -\, x \, \frac{r_{C_i} C_i}{K_{C_i} + C_i}, \qquad i \in S, \tag{9}$$

$$\dot{N} = -\frac{1}{Y_N} \, \dot{x}. \tag{10}$$

At batch termination, the total biomass gain is

$$F(S) = x(t_{\text{stop}}) - x(0).$$

Resource budgets

Let C_{i0} and N_0 be initial stocks. Equation (8) implies the total biomass yield is limited by whichever budget can be fully consumed:

$$F(S) = \min\left(\sum_{i \in S} Y_{C_i} C_{i0}, \; Y_N N_0 \right). \tag{11}$$

Define the nitrogen budget

$$X_{\text{bud}} = Y_N N_0.$$

Thus:

- *C-limited:* $\sum_{i \in S} Y_{C_i} C_{i0} < X_{\text{bud}}$.
- *N-limited:* $\sum_{i \in S} Y_{C_i} C_{i0} \geq X_{\text{bud}}$.

Leave-one-in ($S' = S \cup \{j\}$)

a. C-limited on both S and S'.

$$F(S') = F(S) + Y_{C_j} C_{j0}, \tag{12}$$

$$F' - F = Y_{C_j} C_{j0}. \tag{13}$$

All points lie on a horizontal line.

b. N-limited on both.

$$F(S) = F(S') = X_{\text{bud}} \;\Rightarrow\; F' - F = 0.$$

c. Mixed regime. If S is C-limited but S' crosses into N-limited:

$$F' - F = \min\bigl(Y_{C_j} C_{j0}, \; X_{\text{bud}} - F\bigr). \tag{14}$$

Equation (14) is piecewise linear: a segment of slope -1 as $F \to X_{\text{bud}}$, capped by the intrinsic contribution $Y_{C_j} C_{j0}$.

Interpretation

When nitrogen ultimately limits growth, adding C_j can supply at most the remaining budget $X_{\text{bud}} - F$. This shrinks linearly with F, producing the characteristic straight lines with slope -1 near the N limit and horizontal plateaus elsewhere.

A compact summary is:

$$F' - F = \begin{cases} Y_{C_j} C_{j0}, & \text{C-limited } S, S', \\ 0, & \text{N-limited } S, S', \\ \min\{Y_{C_j} C_{j0}, \; X_{\text{bud}} - F\}, & \text{mixed.} \end{cases}$$

GLOBAL EPISTASIS FROM MANY SPECIES, ONE RESOURCE

1. MODEL DEFINITION

We consider a well-mixed batch culture containing N microbial species that all compete for a single limiting carbon resource C and produce a secreted by-product B in proportion to their resource uptake. The dynamical variables are $C(t)$ (resource concentration), $B(t)$ (by-product concentration), and $x_i(t)$ (biomass of species i).

Each species i is characterized by four parameters: μ_i (maximum specific growth rate), K_M (shared Monod constant), Y_i (biomass yield on carbon), and β_i (by-product stoichiometry). The system evolves according to:

$$\frac{dx_i}{dt} = \mu_i \, \frac{C}{K_M + C} \, x_i \tag{15}$$

$$\frac{dC}{dt} = -\sum_{i=1}^{N} \frac{1}{Y_i} \, \mu_i \, \frac{C}{K_M + C} \, x_i \tag{16}$$

$$\frac{dB}{dt} = \sum_{i=1}^{N} \beta_i \, \frac{1}{Y_i} \, \mu_i \, \frac{C}{K_M + C} \, x_i \tag{17}$$

Growth ceases when the resource is depleted, $C(t_{\text{stop}}) \approx 0$. The quantity of interest is the final by-product $F(S) = B(t_{\text{stop}}; S)$, where S denotes the subset of species present at inoculation.

2. CHANGE OF VARIABLES AND SIMPLIFICATION

Since the Monod factor $C/(K_M + C)$ multiplies every species' growth rate, the dynamics simplify under the reparameterization $d\tau \equiv \frac{C}{K_M+C}\,dt$. Using this substitution, Eqs. (15)–(17) become:

$$\frac{dx_i}{d\tau} = \mu_i x_i, \quad \frac{dC}{d\tau} = -\sum_i \frac{1}{Y_i}\mu_i x_i, \quad \frac{dB}{d\tau} = \sum_i \beta_i \frac{1}{Y_i}\mu_i x_i. \tag{18}$$

These integrate exactly to $x_i(\tau) = x_{i0}\,e^{\mu_i \tau}$. Let τ_f be the effective time at carbon exhaustion ($C(\tau_f) = 0$). Integrating $dC/d\tau$ gives the carbon balance:

$$C_0 = \int_0^{\tau_f} \sum_{i\in S} \frac{1}{Y_i}\mu_i x_i(\tau)\,d\tau = \sum_{i\in S} \frac{x_{i0}}{Y_i}\left(e^{\mu_i \tau_f(S)} - 1\right). \tag{19}$$

We define $U_i(S) \equiv \frac{x_{i0}}{Y_i}\left(e^{\mu_i\tau_f(S)} - 1\right)$ as the total carbon consumed by species i, where $\sum_{i\in S} U_i(S) = C_0$. The final by-product is:

$$F(S) = \sum_{i\in S} \beta_i\,U_i(S). \tag{20}$$

3. TARGET SPECIES ADDITION

Consider a held-out species j and a background S. Define the augmented community $S' = S \cup \{j\}$. We analyze the change in by-product $\Delta F = F(S') - F(S)$.

3.1. Decomposition of the Mean Yield

Define the carbon share of the added species j as $\alpha_j = U_j(S')/C_0$. The remaining carbon is consumed by the background species S, such that $\sum_{k\in S} U_k(S') = C_0(1 - \alpha_j)$. We decompose the mean yield of the augmented community, $\langle\beta\rangle_{S'} = F(S')/C_0$, by splitting the sum:

$$\begin{aligned}
\langle\beta\rangle_{S'} &= \frac{1}{C_0}\left[\sum_{i\in S}\beta_i U_i(S') + \beta_j U_j(S')\right] \\
&= \frac{\sum_{k\in S} U_k(S')}{C_0}\left(\frac{\sum_{i\in S}\beta_i U_i(S')}{\sum_{k\in S} U_k(S')}\right) + \frac{U_j(S')}{C_0}\beta_j \\
&= (1-\alpha_j)\left(\frac{\sum_{i\in S}\beta_i U_i(S')}{\sum_{k\in S} U_k(S')}\right) + \alpha_j\beta_j. \qquad (21)
\end{aligned}$$

3.2. Emergence of Global Epistasis

The term in the parentheses in Eq. (21) represents the internal allocation of the background species S when species j is present. Assuming the addition of species j does not substantially change the internal allocation among species in S ($\langle\beta\rangle_{S',\text{internal}} \approx \langle\beta\rangle_S$), we obtain:

$$\langle\beta\rangle_{S'} \approx (1-\alpha_j)\langle\beta\rangle_S + \alpha_j\beta_j. \tag{22}$$

Calculating the observed change ΔF:

$$\begin{aligned}
F' - F &= C_0\left(\langle\beta\rangle_{S'} - \langle\beta\rangle_S\right) \\
&\approx C_0\left[(1-\alpha_j)\langle\beta\rangle_S + \alpha_j\beta_j - \langle\beta\rangle_S\right] \\
&\approx C_0\alpha_j\beta_j - C_0\alpha_j\langle\beta\rangle_S. \qquad (23)
\end{aligned}$$

Since $F = C_0\langle\beta\rangle_S$, we arrive at the linear relationship:

$$F' - F \approx \alpha_j(\beta_j C_0 - F). \tag{24}$$

CROSS-FEEDING MODEL

We extend the single-resource competition model to include a metabolic cascade. The community now contains two guilds: *producers* (biomass x_p) that consume a primary carbon source C, and *consumers* (biomass x_k) that consume a secondary metabolite C_{cf} leaked by the producers. A fraction p_p of each unit of C taken up by producers is excreted as C_{cf}. Both guilds produce the byproduct B that defines community function. Each producer is parameterized by its maximum growth rate μ_p, biomass yield Y_p, and byproduct stoichiometry β_p; consumers analogously by μ_k, Y_k, β_k. The two guilds use distinct Monod constants K_M (on C) and K_M^{cf} (on C_{cf}). The full dynamics are:

$$\begin{aligned}
\frac{dx_p}{dt} &= \frac{\mu_p C}{K_M + C}x_p \\
\frac{dx_k}{dt} &= \frac{\mu_k C_{cf}}{K_M^{cf} + C_{cf}}x_k \\
\frac{dC}{dt} &= -\sum_p \frac{1}{Y_p}\frac{\mu_p C}{K_M + C}x_p \\
\frac{dC_{cf}}{dt} &= \sum_p p_p\left(\frac{1}{Y_p}\frac{\mu_p C}{K_M + C}x_p\right) - \sum_k \frac{1}{Y_k}\frac{\mu_k C_{cf}}{K_M^{cf} + C_{cf}}x_k \\
\frac{dB}{dt} &= \sum_p \frac{\beta_p}{Y_p}\frac{\mu_p C}{K_M + C}x_p + \sum_k \frac{\beta_k}{Y_k}\frac{\mu_k C_{cf}}{K_M^{cf} + C_{cf}}x_k
\end{aligned} \tag{25}$$

As in the competitive model, simulations are run as batch cultures and function is the accumulated B at carbon depletion.

Density-weighted correlation coefficient

To quantify the degree of linearity in a global epistasis plot we use a density-weighted Pearson correlation coefficient ρ_w between the background function F and the function gain $\Delta F = F' - F$. Across 2^{N-1} background communities, the empirical distribution of F is highly

non-uniform: many background compositions yield similar values of F, producing dense clusters along the F axis (for instance near $F = 0$ when most low-producing backgrounds collapse onto each other), while other regions of F are sampled by relatively few backgrounds. An unweighted Pearson correlation in this setting is dominated by the dense clusters along the F axis. It can therefore miss a linear trend that spans the full range of F, or report spurious linearity when those clusters happen to lie along a line.

To correct for this, each point is weighted by the inverse of the marginal density of F, estimated from the data by a Gaussian kernel density estimate (KDE):

$$w_i \;=\; \frac{1}{\widehat{p}(F_i)}, \tag{26}$$

where $\widehat{p}(F)$ is the KDE of the empirical distribution of $\{F_i\}$. Points in sparse regions of F are upweighted and points in dense regions downweighted, so the resulting correlation reflects linearity across the full range of F rather than within the dominant cluster. Before computing weights we trim the top and bottom 1% of ΔF values to suppress outliers that arise from numerically extinct or near-empty backgrounds.

The density-weighted correlation is then the standard weighted Pearson formula:

$$\rho_w \;=\; \frac{\sum_i w_i(F_i - \bar{F}_w)(\Delta F_i - \overline{\Delta F}_w)}{\sqrt{\sum_i w_i(F_i - \bar{F}_w)^2 \, \sum_i w_i(\Delta F_i - \overline{\Delta F}_w)^2}}, \tag{27}$$

with weighted means $\bar{F}_w = \sum_i w_i F_i / \sum_i w_i$ and $\overline{\Delta F}_w$ defined analogously. With this definition $\rho_w \to -1$ when ΔF depends linearly and monotonically on F across the full sampled range, while $\rho_w \to 0$ signals curvature or saturation that is invisible to a correlation dominated by the densely sampled cluster.

BREAKDOWN OF GLOBAL EPISTASIS

Niche partitioning

The model simulates a batch culture of N species partitioned into two non-interacting guilds, $\mathcal{G}_A$ and $\mathcal{G}_B$, competing for two distinct resources, C_A and C_B. The biomass x_i of species i accumulates via Monod growth on its guild-specific resource:

$$\frac{dx_{i\in\mathcal{G}_A}}{dt} = \mu_i \frac{C_A}{K_M + C_A} x_i, \quad \frac{dx_{i\in\mathcal{G}_B}}{dt} = \mu_i \frac{C_B}{K_M + C_B} x_i \tag{28}$$

Resource depletion is governed by the total uptake within each guild:

$$\frac{dC_A}{dt} = -\sum_{i\in\mathcal{G}_A} \frac{1}{Y_i}\frac{dx_i}{dt}, \quad \frac{dC_B}{dt} = -\sum_{i\in\mathcal{G}_B} \frac{1}{Y_i}\frac{dx_i}{dt} \tag{29}$$

The global community function B represents the total integrated metabolic byproduct yield across both guilds:

$$\frac{dB}{dt} = \sum_{i=1}^{N} \beta_i \frac{1}{Y_i}\frac{dx_i}{dt} \tag{30}$$

The marginal contribution of a focal species, $\Delta F = B_{with} - B_{without}$, depends only on the occupancy of its own niche. Consequently, the global epistasis plot bifurcates: backgrounds consisting solely of the opposing group result in a zero-slope regime (independence), while backgrounds containing the same guild result in a negative-slope regime (competition).

Facilitation

The biomass x_i of each species i follows Monod growth kinetics, defined as:

$$\frac{dx_i}{dt} = \mu_i \frac{C}{K_M + C} x_i \tag{31}$$

where the primary resource C is depleted by the total community uptake:

$$\frac{dC}{dt} = -\sum_{i=1}^{N} \frac{1}{Y_i}\frac{dx_i}{dt} \tag{32}$$

The total community function B is the cumulative byproduct yield, governed by:

$$\frac{dB}{dt} = \sum_{i=1}^{N} \beta_i^{eff} \frac{1}{Y_i}\frac{dx_i}{dt} \tag{33}$$

Synergy is introduced via a conditional yield coefficient β_i^{eff}. If the facilitator (species $j = 0$) is present in the inoculum, the efficiency of all other species $i \neq 0$ is scaled by a synergy boost η:

$$\beta_{i\neq 0}^{eff} = \begin{cases} \beta_i(1+\eta) & \text{if } x_0(t) > \epsilon \\ \beta_i & \text{if } x_0(t) \leq \epsilon \end{cases} \tag{34}$$

This formulation ensures that the marginal fitness contribution ΔF of species 0 is proportional to the total resource processed by the background species, leading to a positive slope in global epistasis scans.

SUPPLEMENTARY FIGURES

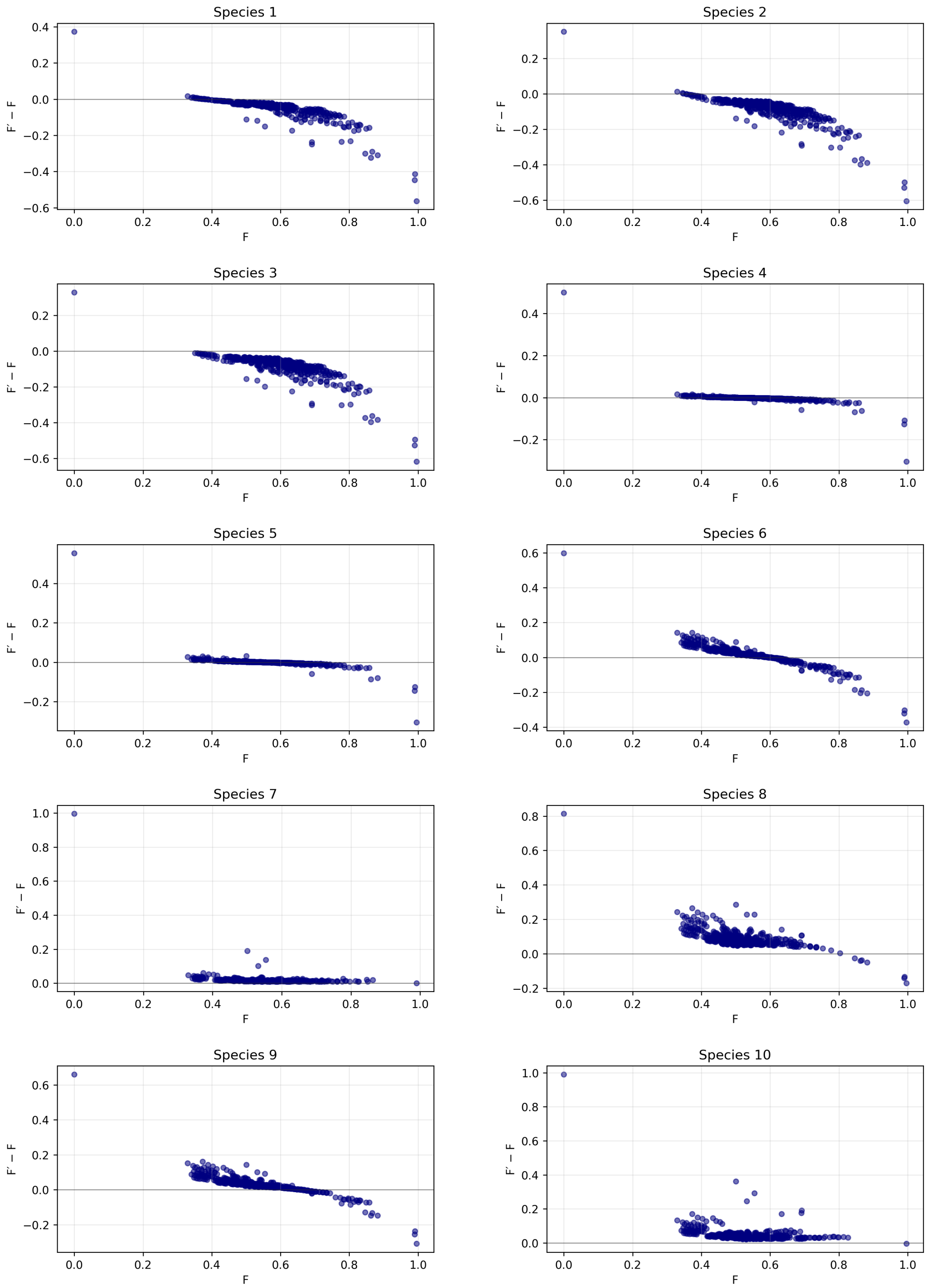

FIG. S1. **Global epistasis for all species in the community used for Figure 3**. The dashed lines are the analytical approximation to the slope given by equation 7 in the main text. Note the point at $F = 0$ is included here but omitted in the main text panels and corresponds to the target species being added to the empty community. The three example species in Figure 3 are 4, 2 and 6 shown in panels b-d respectively.

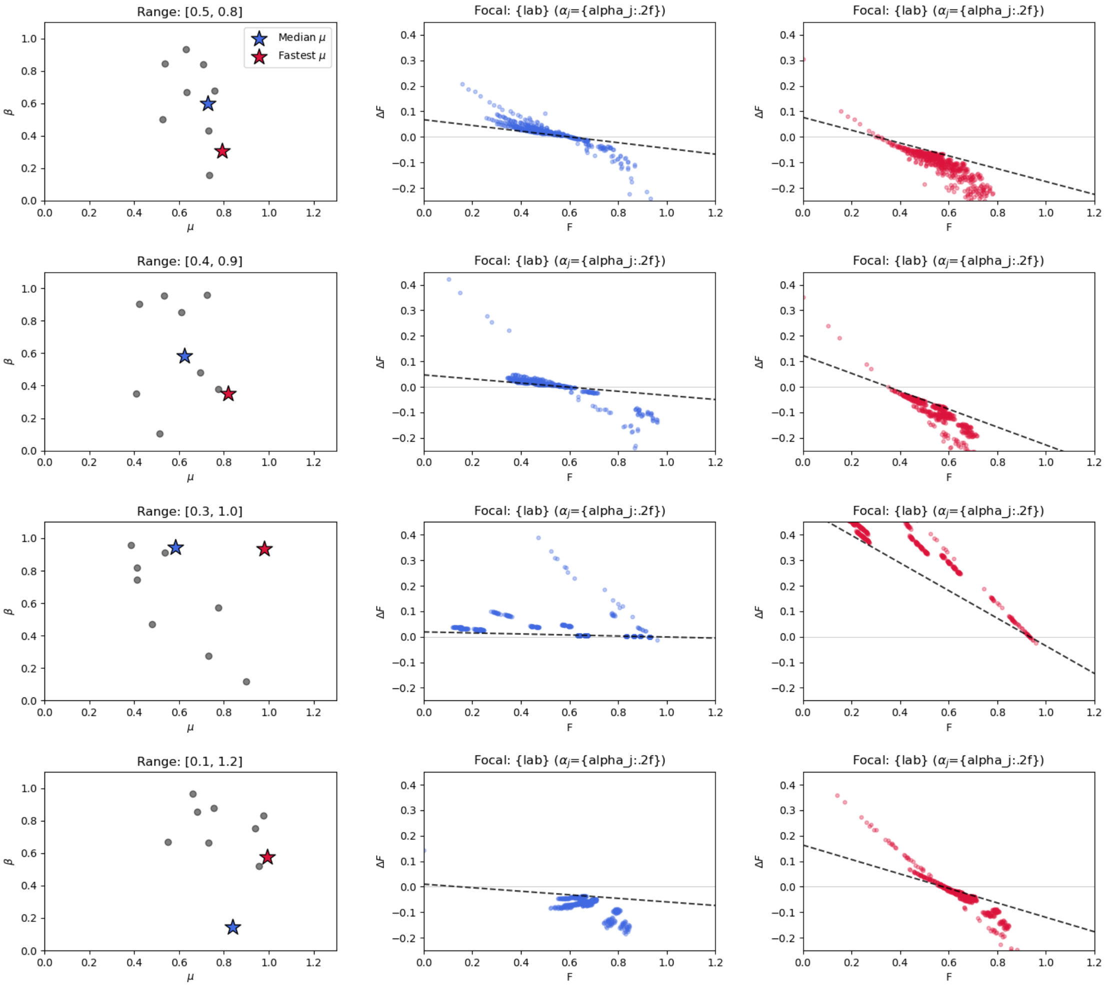

FIG. S2. **Stratification and branching of global epistasis under varying growth rate distributions for 10 species communities.** (Left Column) Phenotype maps showing the distribution of growth rates (μ) and byproduct efficiencies (β) for $N = 10$ species. Red and blue stars indicate the 'Fastest μ' and 'Median μ' focal species, respectively. (Center and Right Columns) Global epistasis (GE) plots for the selected focal species across all $2^{N-1} = 512$ possible backgrounds. **Model Parameters:** Simulations were performed of the model shown in Eqns. 456 with $C_0 = 1.0$, half-saturation constant $K_M = 0.01$, and initial seed biomass $X_0 = 10^{-4}$. Yields (Y) were sampled from a lognormal distribution with a 20% coefficient of variation ($\sigma \approx 0.198$). **Dynamics:** From top to bottom, the range of μ is widened from a tight regime $[0.5, 0.8]$ to an extreme regime $[0.1, 1.2]$. The dashed black lines represent the analytical prediction $\Delta F = \alpha_j(\beta_j C_0 - F)$, where the resource share α_j is determined by a full N-species simulation. **Observations:** As the growth rate range expands, the 'Fastest μ' strain exhibits significant branching, where discrete parallel lineages emerge. These branches represent different competitive regimes where the focal strain either dominates or shares resources with other high-growth competitors, causing the effective α_j to deviate from the community average.

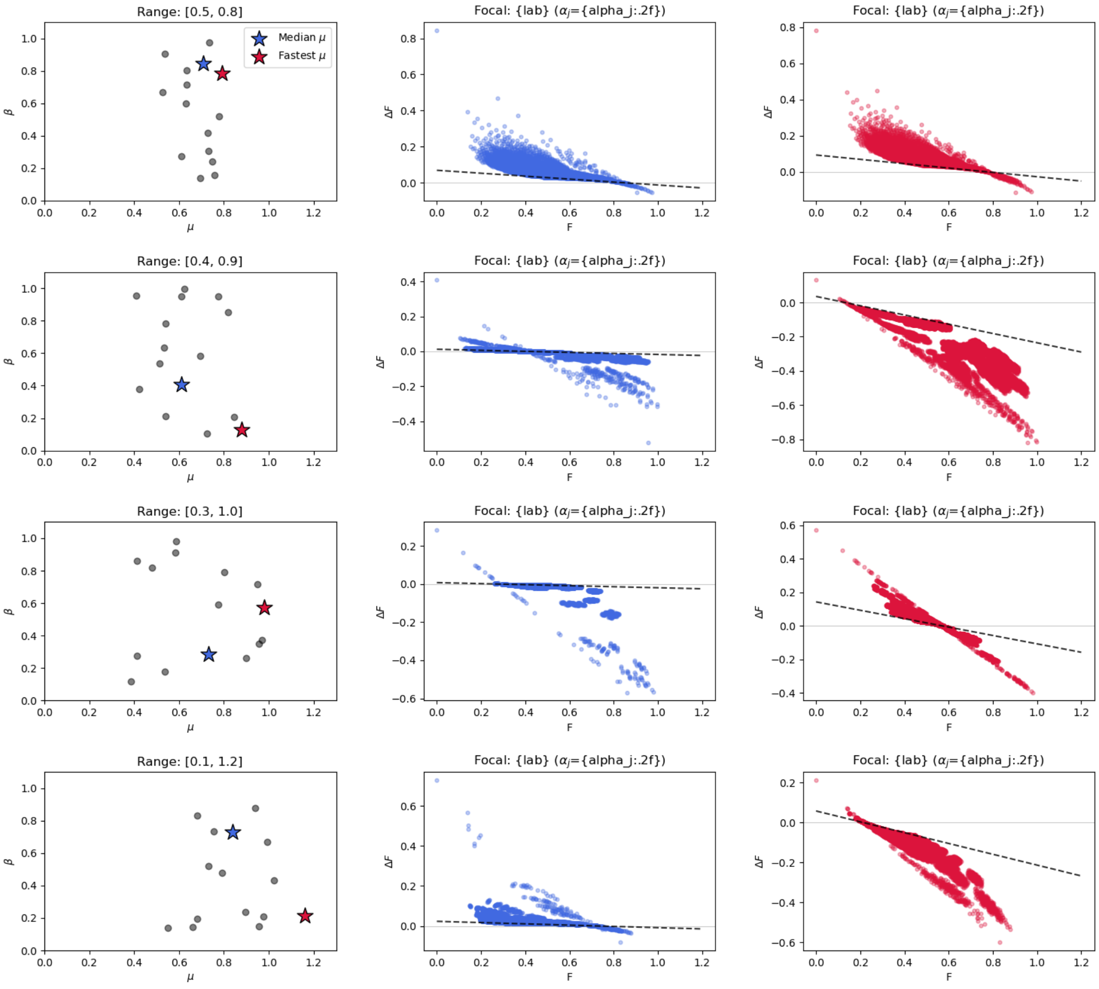

FIG. S3. **Stratification and branching of global epistasis under varying growth rate distributions for 15 species communities.** Identical to Figure S2 but for 15-species communities.

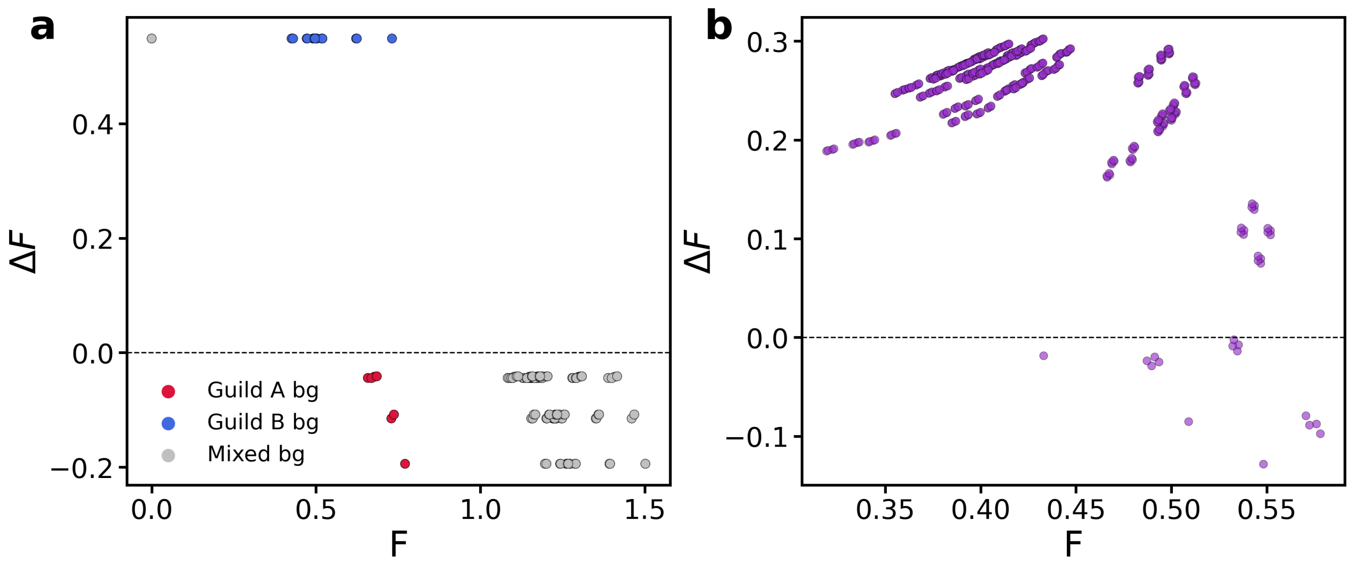

FIG. S4. **Niche partitioning and synergy break global epistasis patterns.** **(a)** Niche partitioning global epistasis plot for a single member of a 10-species community with two guilds that consume separate carbon sources. Points are colored by background composition (Guild A only, Guild B only, or mixed). **(b)** Global epistasis plot for a facilitator strain as described in the appendix. Note the positive slopes in various regions of the ΔF vs. F plane.

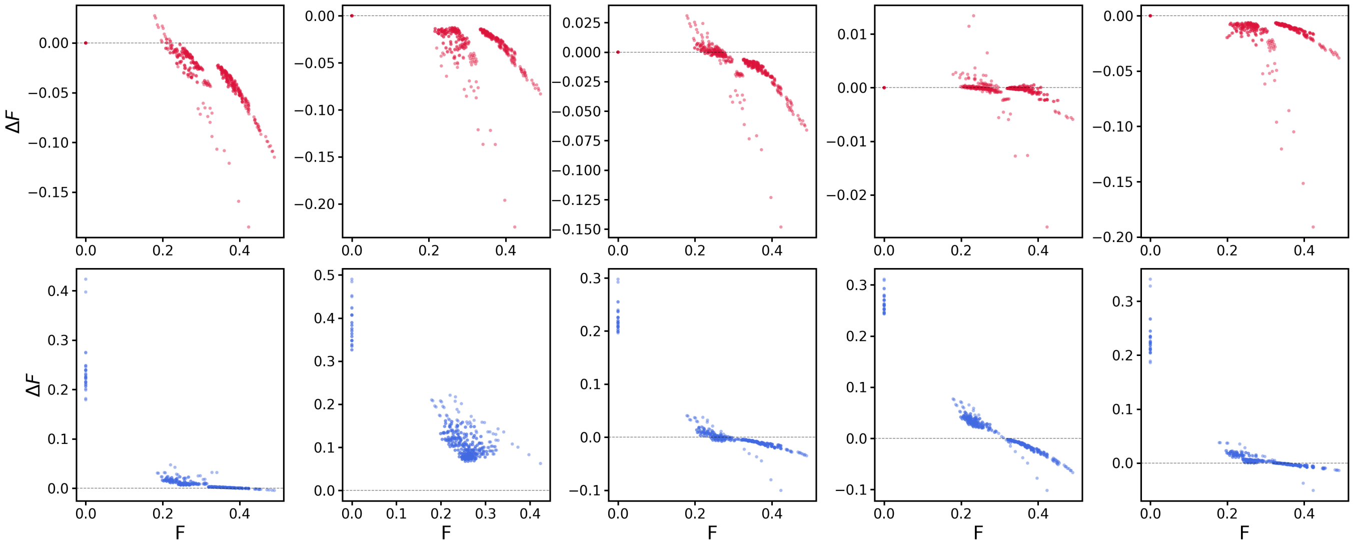

FIG. S5. **Strict cascade global epistasis.** GE plots for a community where the producers (strains degrading C to C_{cf} do not produce byproduct directly ($\beta_i = 0$ for $i \in P$). In this case the intercept for producers is always 0 since they produce no byproduct directly. Note in this case p_i varies across strains unlike the simulations above where it is fixed across all producers.
